# The Draft Genome of Red Lechwe, *Kobus leche leche*

**DOI:** 10.3389/fgene.2020.582638

**Published:** 2020-11-05

**Authors:** Bao Wang, Zhongkai Wang, Jiong Zhou, Wei Liu, Zeshan Lin, Chenzhou Zhang, Guichun Liu, Botong Zhou, Wenting Wan, Ruoping Zhao, Wen Wang, Rasmus Heller, Lei Chen

**Affiliations:** ^1^State Key Laboratory of Genetic Resources and Evolution, Kunming Institute of Zoology, Chinese Academy of Sciences, Kunming, China; ^2^Kunming College of Life Science, University of Chinese Academy of Sciences, Kunming, China; ^3^School of Ecology and Environment, Northwestern Polytechnical University, Xi'an, China; ^4^Section for Computational and RNA Biology, Department of Biology, University of Copenhagen, Copenhagen, Denmark

**Keywords:** *Kobus leche*, red lechwe, genome assembly, phylogenetic relationships, evolution

## Introduction

Red lechwe (*Kobus leche leche*) is the most widespread of three described lechwe (*Kobus leche*) subspecies, belonging to the genus *Kobus* (*Bovidae, Cetartiodactyla, Mammalia*) (IUCNSSC Antelope Specialist Group, 2017). Red lechwe is widely distributed in the wetlands of south-central Africa and particularly adapts to semi-aquatic environments, which is also an embodiment of the ruminants' ecological diversities. Because of its dependence on aquatic and floodplain grasses for feeding, historical red lechwe population sizes could reflect the past distribution of such wetlands (Williamson, 1990) and therefore be an important indicator species for this habitat type. The red lechwe populations have declined drastically over the past years in southern Africa, due to human activities and climate effects (Dipotso and Skarpe, 2006). As a consequence, red lechwe has been categorized in the International Union for Conservation of Nature (IUCN) and Natural Resources Red List and Appendix II of the Convention on International Trade in Endangered Species of Wild Fauna and Flora (CITES) (IUCNSSC Antelope Specialist Group, 2017) as a near-threatened species.

However, the red lechwe genome has not been previously sequenced, which has impeded both biological research and conservation efforts. Herein, we report the first draft genome of red lechwe, which provides a valuable resource not only for ecological and population genetic biology of red lechwe but also further conservation biology studies among endangered mammals.

## Data

**Figure 1:**
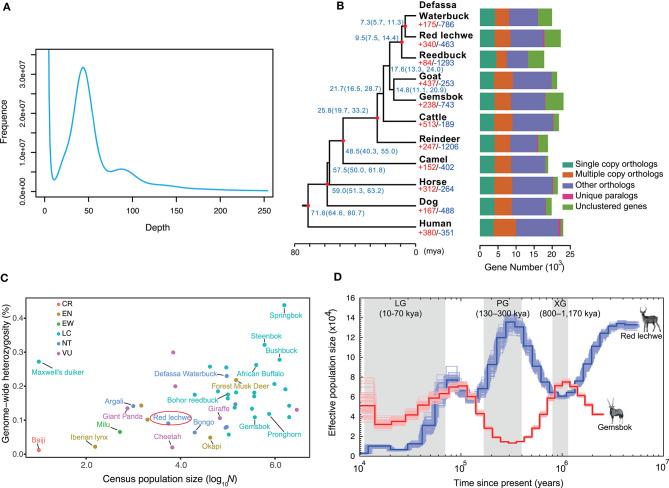
Characteristics of genome assembly for red lechwe and evolutionary analysis. **(A)** Distribution of 17-mer frequency. **(B)** Phylogenetic relationships among 11 mammalian species and orthologous gene families. The proportions of expanded (red) and contracted (blue) gene families are shown at each branch tip. The estimated divergence time is labeled at each node with 95% confidence interval in the brackets. The red circle indicates nodes with fossil records. Bar plots show orthologous gene clusters in each species. **(C)** The distribution of genome-wide heterozygosity against census population sizes for ruminants and some endangered mammals. **(D)** Comparison of demographic history of red lechwe and gemsbok. The blue line indicates red lechwe, and the red line gemsbok.

A whole genome shotgun (WGS) strategy was utilized in this study for genome assembly of red lechwe. In total, 772.34 Gb of raw reads were generated, including 406.44 Gb short-size paired-end reads and 365.90 Gb large-size mate-paired reads. After removing low-quality reads and duplicated reads, about 559.58 Gb of clean reads were retained for genome assembly, with an average coverage of 192.96 x (Supplementary Table 1). Based on *k*-mer analysis, the estimated genome size of red lechwe is 2.92 Gb (Figure 1A). The genome assembly has a total length of 2.77 Gb (accounting for 94.86% of estimated genome size), with a contig N50 length of 61,336 bp and scaffold N50 length of 3,233,651 bp, which are compared with the closely related species Defassa waterbuck (*Kobus ellipsiprymnus*, GenBank: GCA_006410655.1) and goat (*Capra hircus*, GenBank: GCA_002263795.2) (Table 1). The guanine-cytosine (GC) content of the assembled genome is similar to that of cattle (UMD3.1) and goat genome (ARS1) (Supplementary Figure 1).

**Table 1:** Summary of the red lechwe genome and annotation.

	**Red lechwe**				**Defassa waterbuck** **(GenBank: GCA_006410655.1)**				**Goat** **(GenBank: GCA_001704415.1)**			
	**Contig**		**Scaffold**		**Contig**		**Scaffold**		**Contig**		**Scaffold**	
	**Size (bp)**	**Number**	**Size (bp)**	**Number**	**Size (bp)**	**Number**	**Size (bp)**	**Number**	**Size (bp)**	**Number**	**Size (bp)**	**Number**
**Genome assembly**												
N90	9,688	54,037	360,664	1,108	3,860	149,509	133,320	4,237	23,439	28,921	28,168	660
N80	20,634	34,876	1,046,549	680	7,630	100,313	300,206	2,859	40,885	20,521	51,332,696	26
N70	32,643	24,340	1,757,042	477	11,576	71,498	452,685	2,082	58,183	15,103	66,011,198	21
N60	46,061	17,267	2,554,374	348	15,878	51,453	605,984	1,529	75,811	11,121	71,784,255	17
N50	61,336	12,092	3,233,651	252	20,724	36,430	779,552	1,111	96,009	8,020	87,277,232	13
Longest (bp)	876,794		16,823,847		285,199		4,602,372		1,160,130		157,403,528	
Total number	152,596		57,712		335,525		88,848		76,586		29,907	
Total size (bp)	2,748,823,911		2,771,256,083		2,725,194,340		2,895,340,485		2,649,649,435		2,922,813,246	
**Gene prediction and annotation**												
Number of protein-coding genes			22,375		19,994				21,343			
Average gene length (kb)			30.26		38.08				39.89			
Average cds length (bp)			1,432.09		1,586.88				1,536.10			
Average number of exons per gene			8.54		9.50				8.90			
Average length of exon (bp)			167.72		167.00				172.80			
Average length of intron (kb)			3.82		3.88				4.2			

To evaluate the completeness of the red lechwe genome, three approaches were utilized. First, BUSCO analysis (Waterhouse et al., 2018) showed that 3,820 (93.08%) genes had complete gene coverage (including 1.85% duplicated ones), only 139 (3.39%) were fragmented, and 145 (3.53%) were missing based on 4,104 single-copy ortholog groups of “mammalia_odb9” dataset (Supplementary Table 2). Second, 99.47% of the short-size paired-end reads were successfully mapped into the assembled genome. Finally, syntenic relationships showed that about 2.25 Gb (81.00%) of the assembled genome could be aligned to the goat reference genome (ARS1) with high confidence (–m 0.01) (Supplementary Figure 2). All these results suggest a well-assembled genome of red lechwe with high completeness and continuity.

A total of 1.31 Gb non-redundant repeat elements were identified, accounting for 47.15% of the red lechwe genome (Supplementary Table 3). Of these elements, long interspersed nuclear element (LINE) repeats were the most predominant, accounting for 34.30% of the whole genome (19.93% for BovB subtypes). Using a combination of *de novo* and homolog-based approaches, we predicted a total of 22,375 protein-coding genes (Table 1, Supplementary Table 4, and Supplementary Figure 3). Of these genes, 19,552 (87.38%) were successfully annotated by at least one database, including InterPro (Mitchell et al., 2019), GO (The Gene Ontology Consortium, 2019), KEGG (Kanehisa and Goto, 2000), UniProt/SwissProt (UniProt Consortium, 2019), and TrEMBL (Bairoch and Apweiler, 2000) (Supplementary Table 5).

To estimate species-specific and shared genes in the red lechwe compared with 10 other mammalian species, OrthoMCL (Li et al., 2003) was used to define the orthologous genes and revealed that red lechwe has 14,081 of 19,373 gene families among the mammalian species (Figure 1B and Supplementary Table 6). A genome-wide set of 2,766 one-to-one orthologous gene clusters were used to reconstruct the genome-wide phylogenetic tree. As expected, red lechwe was genetically closest to the Defassa waterbuck (*Kobus ellipsiprymnus*), both of which belong to the *Kobus* genus. The divergence time between red lechwe and defassa waterbuck was estimated at 7.3 (95% CIs, 5.7–11.3) million years ago (mya) (Figure 1C), which is considerably older than previous estimates based on mtDNA and/or fossils (Hernandez-Fernandez and Vrba, 2005; Bibi, 2013). This discrepancy warrants further investigation, as it could be compatible with continued gene flow between the two species after initial divergence. Through gene family analysis, red lechwe has 340/463 significantly expanded/contracted gene families, respectively. The expanded families were enriched in 34 GO categories (Supplementary Table 7), including transporter activity and amine metabolism, while the contracted families were enriched in 23 categories, including microtubule-based movement and cell adhesion (Supplementary Table 8). For the positive selection analysis, a total of 38 and 28 positively selected genes were identified in the red lechwe lineage and *Kobus* lineage (Supplementary Tables 9, 10). Furthermore, we detected 460 and 324 rapidly evolving genes in red lechwe and *Kobus* linage (Supplementary Tables 11, 12). Rapidly evolving genes in red lechwe lineage were mainly enriched in regulation of autophagy, endothelial cell proliferation, metabolic process, and nervous system (Supplementary Table 13), while rapidly evolving genes in the *Kobus* lineage were mainly enriched in nervous system, bone resorption and remodeling, bicarbonate transport, and immunity (Supplementary Table 14).

Based on the assembly, we assessed the heterozygosity rate of red lechwe at 0.90%, which is even lower than giant panda (Li et al., 2010). Among the sequenced ruminants, red lechwe has relatively lower heterozygosity ratio and smaller census population sizes (Figure 1C and Supplementary Table 5), arousing the urgency of their conservation studies. Moreover, we compared the inferred effective population size of red lechwe with that of gemsbok, which is a notoriously drought-tolerant African antelope living in deserts (Farre et al., 2019). Until the end of the Penultimate Glaciation (PG, 130–300 thousand years ago, kya), the effective population sizes of these two species show remarkably consistent opposite trends (Figure 1D). Hence, it is tempting to speculate that the population sizes of these two species, placed at opposing extremes of the drought tolerance continuum, have tracked the cycles of pluvials and interpluvials in tropical Africa during the latter half of the Pleistocene (Lorenzen et al., 2012). During the last 100 kya, population size decreased for both species, consistent with a ruminant-wide decline during this period, possibly related to an increased human ecological footprint (Chen et al., 2019). We therefore propose that the population size of these African antelopes could have been shaped by different external factors at different periods in their prehistory.

## Materials and Methods

### Sample Collection, Library Construction, Sequencing, and Quality Control

The sample from a male red lechwe was generously provided by Copenhagen Zoo. It can be traced back four generations in captivity, and the origin of its wild ancestor could not be verified (Supplementary Figure 4). Genomic DNA of the red lechwe was isolated from adult muscle tissue following the protocol of DNeasy Blood & Tissue kit (Qiagen, USA). Whole genome shotgun sequencing strategy was applied for sequencing, and a series of DNA libraries (300, 500, 800 bp, 2, 5, 8, and 15 kb) were constructed following the standard protocol provided by Illumina (San Diego, CA, USA). To construct small-insert libraries (300, 500, and 800 bp) for Illumina sequencing, genomic DNA was randomly sheared into 180–800 bp fragments using a Covaris S2 sonicator (Covaris, Woburn, MA, USA), end-repaired, A-tailed, and ligated to Illumina paired-end adapters (Illumina, San Diego, USA) using TruSeq DNA Sample Preparation Kit. The ligated fragments were selected at 300, 500, and 800 bp on an agarose gel and amplified by PCR to yield the corresponding short-insert libraries. To construct long-insert libraries (2, 5, 8, and 15 kb), genomic DNA was fragmented using a Hydroshear system (Digilab, Marlborough, MA, USA). The DNA fragments were end repaired using biotinylated nucleotide analogs, fragments of the desired size were gel purified at 2, 5, 8, and 15 kb.

All libraries were sequenced on an Illumina HiSeq 2500 platform (Illumina; CA, USA) to generate 2 × 150 bp paired-end reads. For the Illumina sequencing data, data quality control of raw reads was performed using FastQC v0.11.5 (Andrews, 2010). The reads were filtered out as follows: (1) reads with mean Phred score less than 10 for 30% of bases; (2) reads with more than 10% of Ns; (3) reads with an adapter sequence of >10 bp; (4) paired-ends reads that had completely identical sequences (considered as the products of PCR duplicates).

### Estimation of Genome Size and Genome Assembly

Small-insert size libraries were employed to estimate genome size by *k*-mer analysis (Liu et al., 2013). The genome size was estimated by the formula *G* = *k*_num/*k*_depth, where *G* represents genome size, *k*_num represents the total number of *k*-mers, and *k*_depth is the average depth of *k*-mers. In this study, all clean reads from small-insert size libraries were employed to conduct the 17-mer analysis.

Platanus assembler (v1.2.4) (Kajitani et al., 2014) was used to assemble the red lechwe genome with default parameters. First, contig assembly was performed using *platanus assemble* with small-insert size libraries as input (300, 500, and 800 bp). Then, scaffold assembly was performed using *platanus scaffold* with insert-size libraries ≥ 500 bp as input. Finally, the gaps remaining in the generated scaffolds were filled using *platanus gap_close* with 300 and 500 bp insert-size libraries as input.

### Quality Evaluation of Assembled Genome

The quality of the lechwe genome assembly was evaluated using three approaches. First, we performed a BUSCO v3.0.2 (Waterhouse et al., 2018) analysis based on 4,104 single-copy ortholog groups in mammalia_odb9 database. Then, we mapped the short-size libraries onto the red lechwe draft genome using BWA 0.7.17-r1188 software (Li and Durbin, 2009) with BWA-mem default parameters. Finally, we compared the syntenic relationships of the red lechwe draft genome and domestic goat reference genome (ARS1, GenBank: GCA_001704415.1) (Bickhart et al., 2017), a high-quality reference genome. Syntenic relationships were constructed using the program LAST (Kielbasa et al., 2011).

### Genome Annotation of Repetitive Elements and Protein-Coding Genes

#### Repetitive Elements Annotation

We identified repeat elements using a combination of homology-based and *de novo* approaches across the red lechwe assembly. For the homology-based approach, transposable elements were identified using RepeatMasker open-4.0.5 (Tarailo-Graovac and Chen, 2009) and RepeatProteinMask (included in RepeatMasker) against known sequences within the DNA repeat database (RepBase version 16.02) (Bao et al., 2015) at the DNA level and protein level, respectively. For *de novo* prediction, RepeatModeler (version 1.0.4) was used to construct a *de novo* repeat library, and then RepeatMasker was used to identify repeats against the constructed library. Tandem repeats were annotated with Tandem Repeats Finder software (TRF, v4.07b) (Benson, 1999). Finally, all the repeat elements identified above were combined according to their intersected coordinates in the genome.

#### Protein-Coding Genes Annotation

We used a homology-based approach and a *de novo* prediction to annotate protein-coding genes. For the homolog-based approach, protein-coding sequences from three different species *Bos taurus* (UMD3.1), *Homo sapiens* (GRCh38), and *Ovis aries* (Oar_v3.1) (downloaded from Ensembl database release 94) were mapped against the repeat-masked red lechwe genome using TblastN with an *E* value cutoff of 1e−5. Then, GeneWise (version wise2.2.0) (Birney et al., 2004) was used to predict gene models with the aligned sequences as well as the corresponding query proteins. For *de novo* annotation, SNAP v2006-07-28 (with default parameters) (Korf, 2004), glimmerHMM v3.0.2 (with –*f* – *g* parameters) (Majoros et al., 2004), Augustus v2.5.5 (with—*uniqueGeneId* = true—*noInFrameStop* = true—*gff*3 = on parameters) (Stanke et al., 2008) and GENSCAN (version 1.0, with default parameters) (Burge and Karlin, 1998) were utilized with the repeat-masked genome. Finally, EVidenceModeler software (EVM, version 1.1.1, with—*segmentSize* 5,000,000—*overlapSize* 10,000 parameters) (Haas et al., 2008) was used to combine all the predicted gene models to form a comprehensive, non-redundant gene set. The combined predicted genes were removed if they contained (i) no start codon, (ii) no stop codon, or (iii) one or more internal stop codons.

### Functional Annotation of Protein-Coding Genes

Functions of genes were assigned according to best hits derived from the alignments to proteins from KEGG, TrEMBL, and SwissProt databases using BLASTP with an *E* value cutoff of 1e−5. Pfam (Finn et al., 2014), PRINTS (Attwood et al., 2003), SMART (Letunic et al., 2012), and ProDom (Servant et al., 2002) databases were employed to search known motifs and domains in the red lechwe genome using InterProScan v4.8 (Jones et al., 2014). The genes annotated in at least one database mentioned above were considered to show functional evidence.

### Reconstruction of Phylogenetic Relationships

The protein coding genes from 11 mammalian species (Supplementary Table 6), downloaded from Ensembl database and from our previous work (Chen et al., 2019) were used to identify orthologous gene clusters. The longest transcript for each species was chosen to represent each gene, with amino acids >30 retained for subsequent analysis. OrthoMCL v2.0.9 (Li et al., 2003) was utilized to identify clusters with all-to-all BLASTP results as input with an *e* value cutoff of 1e−5 and a Markov chain clustering default inflation parameter. The phylogenetic tree was reconstructed using 2,766 single-copy gene orthologs from the above-mentioned 11 mammalian species, which were aligned using MUSCLE v3.8.31 (Edgar, 2004) and then concatenated into a supergene for each species. Four-fold degenerate sites identified within each supergene were used to reconstruct a phylogenetic tree using RAxML (version 8.2.12) (Stamatakis, 2014) with the GTR+G+I model. The divergence time was estimated using MCMCTREE with a correlated rate clock model implemented in the Phylogenetic Analysis by Maximum Likelihood (PAML) package (Yang, 2007). Calibration times were achieved from TimeTree database (http://www.timetree.org/) and fossil evidence (human-other 64.7–80.9 mya; horse-dog 50.3–62.9 mya; camel-other 33.9–58.8 mya; reindeer-other 17.2–34.4 mya) (Gingerich, 1989; Fox and Scott, 2011; Kumar et al., 2016). The analysis was run twice to ensure the convergence of results.

### Gene Family and Positive Selection Analysis

Based on the OrthoMCL results, CAFÉ (version 4.2.1) (De Bie et al., 2006) was applied to identify gene families that have undergone expansion and contraction with a *p-*value cut-off of 0.05. We further used PAML (Yang, 2007) to detect positively selected genes in the lineage of red lechwe and *Kobus* lineage (including red lechwe and waterbuck). Briefly, a conserved genome synteny methodology was used to determine orthologous gene sets, as described in our previous work (Chen et al., 2019). Positive selection signals in genes were detected using the branch-site model. A likelihood ratio test (LRT) was conducted to compare ratios of non-synonymous to synonymous in selected lineage and the background lineage and determine positive selected genes (*p* < 0.05, according to chi-square statistics). Rapidly evolving genes (REGs) were identified using the branch model. Genes with a *p* < 0.05 and higher ω value of selected lineages than the background branches were considered as REGs.

### Genome-Wide Heterozygosity and Demographic History Estimation

To estimate the heterozygosity of red lechwe and other mammals, raw reads were obtained from National Center for Biotechnology Information (NCBI) and filtered then mapped to the soft-masked genome using BWA-mem (Li and Durbin, 2009). Single nucleotide polymorphisms (SNPs) were called using SAMtools v1.3.1 (Li et al., 2009) with the parameters “mpileup -q 20 -Q 20,” and only biallelic SNPs were included using BCFtools v1.9 (Li, 2011). The proportion of polymorphic sites over the total number of sites is identified as the genome-wide heterozygosity. To reconstruct the demographic history of red lechwe and gemsbok, the genotypes with a depth of coverage less than a third of the average depth or greater than twice were further filtered. The nucleotide substitution rate (per site per year) was calculated using r8s (v1.70) (Sanderson, 2003), which is 1.01e−09 and 2.13e−09 for red lechwe and gemsbok, respectively. Based on the generation time of 6.4 years (IUCNSSC Antelope Specialist Group, 2017) and 7.1 years (Chen et al., 2019) for red lechwe and gemsbok, respectively, pairwise sequentially Markovian coalescent (PSMC, v0.6.5-r67) (Li and Durbin, 2011) analysis was performed to infer the dynamics of effective population sizes using the following parameters: –*N*25 – *t*15 – *r*5 – *b* – *p* “4 + 25 ^*^ 2 + 4 + 6” (*N*, maximum number of iterations; *t*, maximum 2N0 coalescent time; *r*, initial theta/rho ratio; *b*,: bootstrap; and *p*, pattern of parameters).

## Data Availability Statement

The datasets generated for this study can be found in the BioProject database under the accession number PRJNA599106.

## Ethics Statement

The animal study was reviewed and approved by Animal Care and Use Committee of Kunming Institute of Zoology Animal Care and Use Committee of Northwestern Polytechnical University.

## Author Contributions

LC and RH conceived and supervised the project. GL, WWan, and RZ prepared the samples and carried out the experiments. BW, ZW, JZ, WL, ZL, and CZ performed bioinformatics analyses. BW, LC, and WWang wrote and edited the manuscript. All authors have read and approved the final manuscript.

## Conflict of Interest

The authors declare that the research was conducted in the absence of any commercial or financial relationships that could be construed as a potential conflict of interest.
