# Supplementary material for: The Draft Genome of Red Lechwe, Kobus leche leche
Source: Front Genet. 2020 Nov 5;11:582638. doi: 10.3389/fgene.2020.582638 (PMC7674670; doi:10.3389/fgene.2020.582638)
Supplement: Supplementary file 1 [file Data_Sheet_1.zip › Suppl. figures & Table S1-S6.DOCX]

# Supplementary Figures


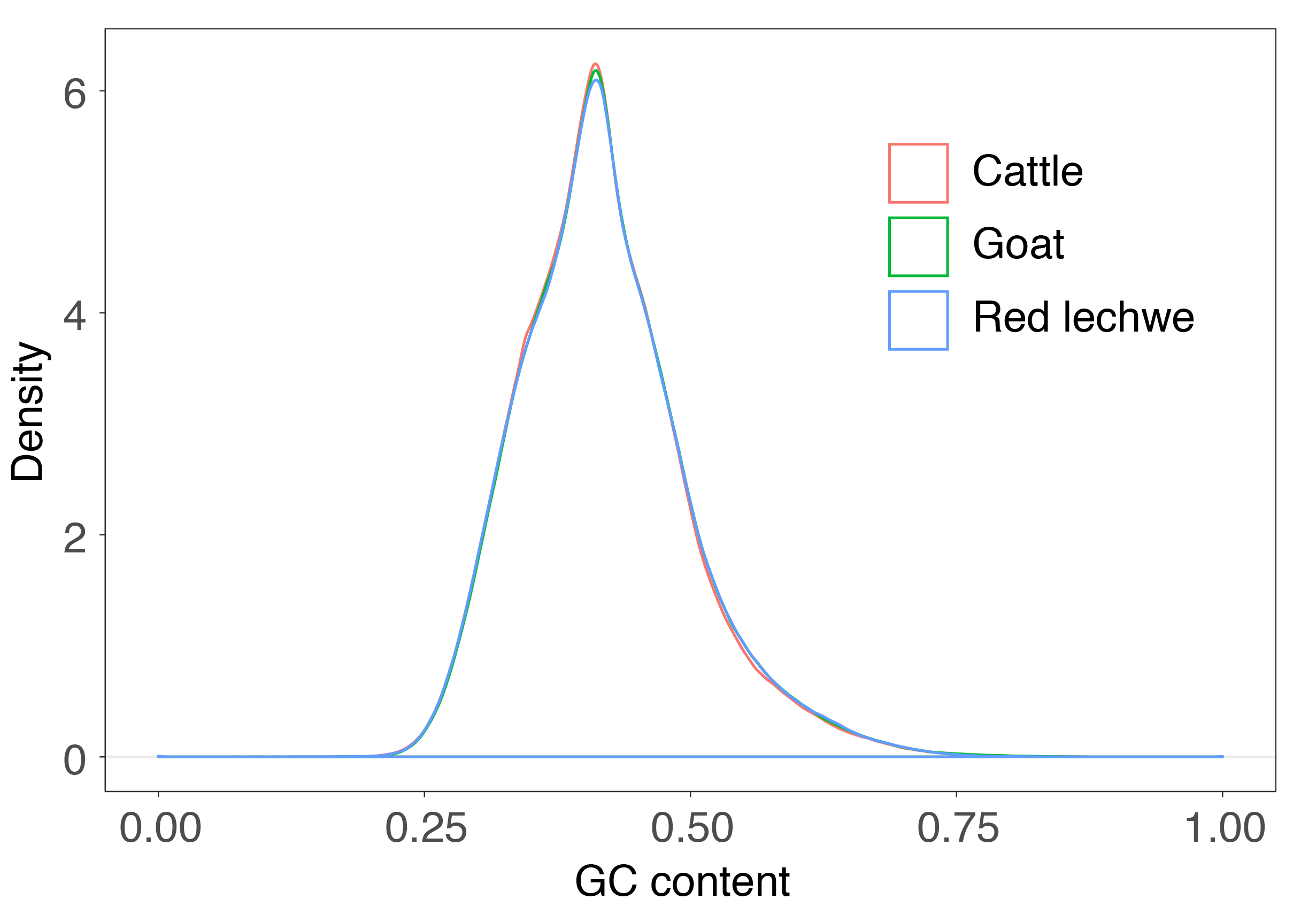


**Supplementary Figure S1.** **The distribution of GC content across genomes of the red lechwe, goat and cattle**. The GC content was calculated by 500-bp non-overlapping sliding windows.

**
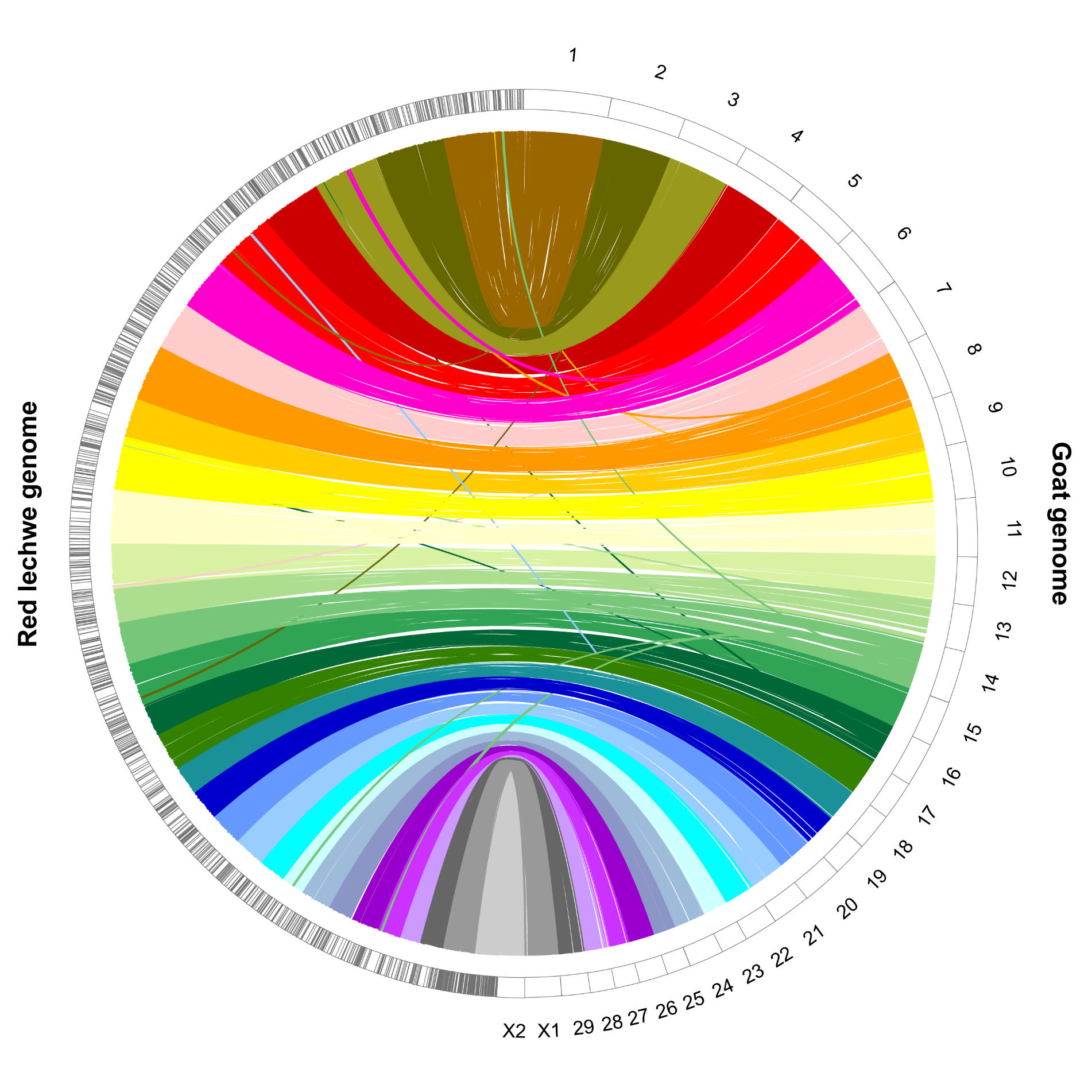
**

**Supplementary Figure S2. Syntenic relationship of the red lechwe genome with the goat genome (ARS1).** Synteny block matching of the red lechwe genome against the goat genome (ARS1) using LAST. Scaffolds less than 100 kb in the red lechwe genome were discarded.


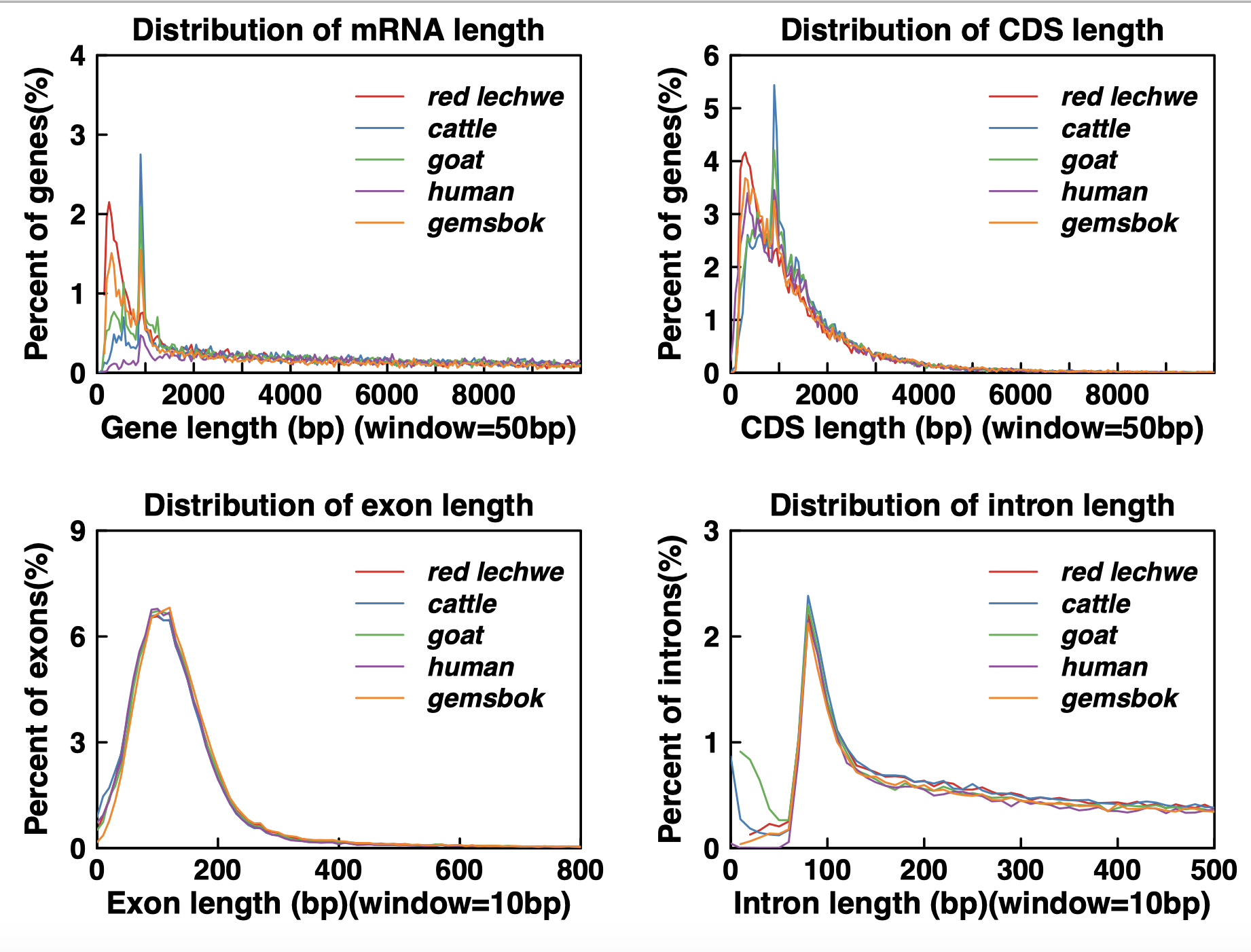


**Supplementary Figure S3. Quality comparison of protein-coding genes**. The distribution of mRNA, CDS, exon, and intron among these species were shown. The species include red lechwe, cattle, goat, human and gemsbok.


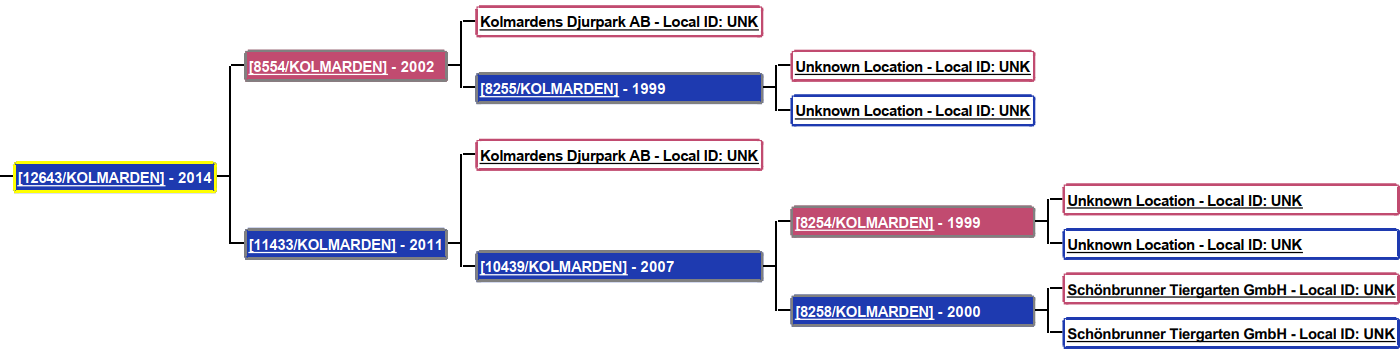


**Supplementary Figure S4.** The pedigree of the sequenced red lechwe

# Supplementary Tables

## Supplementary Table S1. Statistics summary of sequenced reads

| **Library** | **Insert size** | **Raw reads (Gb)** | **Coverage**  **(ⅹ)** | **Clean reads (Gb)** | **Coverage**  **(ⅹ)** |
| --- | --- | --- | --- | --- | --- |
| DSL-1 | 300 bp | 83.13 | 28.66 | 69.37 | 23.92 |
| DSL-4 | 300 bp | 97.31 | 33.56 | 80.63 | 27.80 |
| DSL-6 | 500 bp | 81.37 | 28.06 | 71.10 | 24.52 |
| DSL-8 | 500 bp | 73.41 | 25.31 | 65.85 | 22.71 |
| DSL-10 | 800 bp | 71.22 | 24.56 | 61.58 | 21.24 |
| MSL1 | 2 kb | 88.45 | 30.50 | 69.20 | 23.86 |
| MSL2 | 5 kb | 86.83 | 29.94 | 69.30 | 23.90 |
| MSL3 | 8 kb | 108.37 | 37.37 | 40.60 | 14.00 |
| MSL4 | 15 kb | 82.25 | 28.36 | 31.95 | 11.02 |
|  |  | 772.34 | 266.32 | 559.58 | 192.96 |

## Supplementary Table S2. BUSCO results of the red lechwe genome.

|  | **Proteins** | **Percent (%)** |
| --- | --- | --- |
| Complete BUSCOs (C) | 3,820 | 93.08 |
| Complete and single-copy BUSCOs (S) | 3,744 | 91.23 |
| Complete and duplicated BUSCOs (D) | 76 | 1.85 |
| Fragmented BUSCOs (F) | 139 | 3.39 |
| Missing BUSCOs (M) | 145 | 3.53 |
| Total BUSCO groups searched | 4,104 | 100.00 |

## Supplementary Table S3. Statistics of repeat elements

|  | Repbase TEs | | TE proteins | | De novo | | Combined TEs | |
| --- | --- | --- | --- | --- | --- | --- | --- | --- |
| Type | Length (bp) | % in genome | Length (bp) | % in genome | Length (bp) | % in genome | Length (bp) | % in genome |
| DNA | 48,226,989 | 1.74 | 6,861,874 | 0.25 | 29,590,852 | 1.07 | 56,989,496 | 2.06 |
| LINE | 760,106,148 | 27.41 | 549,726,527 | 19.82 | 780,395,027 | 28.14 | 951,026,243 | 34.30 |
| SINE | 209,639,933 | 7.56 | 0 | 0 | 24,982,901 | 0.90 | 211,593,309 | 7.63 |
| LTR | 130,017,177 | 4.69 | 12,767,340 | 0.46 | 77,519,978 | 2.80 | 141,593,247 | 5.11 |
| Unknown | 424,643 | 0.015 | 0 | 0 | 40,292,640 | 1.45 | 40,698,931 | 1.47 |
| Total | 1,136,559,565 | 40.99 | 569,110,673 | 20.52 | 1,056,418,357 | 38.10 | 1,307,596,684 | 47.15 |

## Supplementary Table S4. Statistics of annotated gene features

| **Gene set** | **Numbers** | **Average gene length (bp)** | **Average cds length (bp)** | **Average exon number** | **Average exon length (bp)** | **Average intron length (bp)** |
| --- | --- | --- | --- | --- | --- | --- |
| **Human** | 19,892 | 54,929.22 | 1,455.35 | 8.49 | 171.48 | 5,630.60 |
| **Cattle** | 19,994 | 38,081.06 | 1,586.88 | 9.50 | 167.00 | 3,877.63 |
| **Goat** | 21,343 | 37,446.88 | 1,536.10 | 8.89 | 172.79 | 4,221.38 |
| **Gemsbok** | 23,125 | 46,718.54 | 1,520.79 | 8.51 | 178.65 | 6,016.29 |
| **Red lechwe** | 22,375 | 30,260.85 | 1,432.09 | 8.54 | 167.72 | 3,824.25 |

## Supplementary Table S5. Functional annotation of protein-coding genes using different databases.

|  | **Number** | **Percent (%)** |
| --- | --- | --- |
| InterProScan | 17,686 | 79.04 |
| GO | 13,971 | 62.44 |
| KEGG | 14,800 | 66.15 |
| Swissprot | 18,583 | 83.05 |
| TrEMBL | 19,520 | 87.24 |
|  | 19,552 | 87.38 |

## Supplementary Table S6. Reference genomes used during the analysis

| **Species** | **Common name** | **Version** | **Source** |
| --- | --- | --- | --- |
| ***Homo sapiens*** | Human | GRCh38 | ftp://ftp.ensembl.org/pub/release-94/fasta/homo_sapiens/dna/ |
| ***Canis familiaris*** | Dog | CanFam3.1 | ftp://ftp.ensembl.org/pub/release-94/fasta/canis_familiaris/dna/ |
| ***Capra Hircus*** | *Goat* | ARS1 | ftp://ftp.ensembl.org/pub/release-94/fasta/capra_hircus/dna/ |
| ***Bos taurus*** | Cattle | UMD3.1 | ftp://ftp.ensembl.org/pub/release-94/fasta/bos_taurus/dna/ |
| ***Camelus bactrianus*** | Camel | MBC_1.0 | ftp://ftp.ncbi.nlm.nih.gov/genomes/all/GCF/000/767/855/GCF_000767855.1_Ca_bactrianus_MBC_1.0 |
| ***Equus caballus*** | Horse | EquCab3.0 | ftp://ftp.ensembl.org/pub/release-95/fasta/equus_caballus/dna/ |
| ***Rangifer tarandus^1^*** | Reindeer | - | http://gigadb.org/dataset/100370 |
| ***Oryx gazelle^2^*** | Gemsbok | GCA_006410575.1 | https://www.ncbi.nlm.nih.gov/assembly/GCA_006410575.1/ |
| ***Redunca redunca^2^*** | Bohor reedbuck | GCA_006410935.1 | https://www.ncbi.nlm.nih.gov/assembly/GCA_006410935.1 |
| ***Kobus ellipsiprymnus^2^*** | Defassa waterbuck | GCA_006410655.1 | https://www.ncbi.nlm.nih.gov/assembly/GCA_006410655.1 |
| ***Kobus leche ssp. leche^3^*** | Red lechwe | - | - |

^1^ Genome annotation was from *Li et al. (2017)*

^2^ Genome annotations were from *Chen et al. (2019)*

^3^ Genome annotation were from this study.

## Supplementary Table S7. GO enrichment analysis of the expanded gene families in the red lechwe genome

## Supplementary Table S8. GO enrichment analysis of the contracted gene families in the red lechwe genome

## Supplementary Table S9. Positively selected genes in red lechwe lineage

## Supplementary Table S10. Positively selected genes in *Kobus* lineage

## Supplementary Table S11. Rapidly evolving genes in the red lechwe lineage

## Supplementary Table S12. Rapidly evolving genes in Kobus lineage

## Supplementary Table S13. GO enrichment analysis of rapidly evolving genes in red lechwe lineage

## Supplementary Table S14. GO enrichment analysis of rapidly evolving genes in *Kobus* lineage

## Supplementary Table S15. Genome-wide heterozygosity estimates and estimated census population size for red lechwe and other mammals

# Reference:

1. Li, Z. P. *et al.* Draft genome of the reindeer (Rangifer tarandus). *Gigascience* **6**, doi:10.1093/gigascience/gix102 (2017).

2. Chen, L. *et al.* Large-scale ruminant genome sequencing provides insights into their evolution and distinct traits. *Science* **364**, doi:10.1126/science.aav6202 (2019).
